# DNA Tumor Viruses and Cell Metabolism

**DOI:** 10.1155/2016/6468342

**Published:** 2016-02-29

**Authors:** Muhammad Mushtaq, Suhas Darekar, Elena Kashuba

**Affiliations:** ^1^Department of Microbiology, Tumor and Cell Biology (MTC), Karolinska Institute, 17177 Stockholm, Sweden; ^2^R.E. Kavetsky Institute of Experimental Pathology, Oncology and Radiobiology, NASU, Kyiv 03022, Ukraine

## Abstract

Viruses play an important role in cancerogenesis. It is estimated that approximately 20% of all cancers are linked to infectious agents. The viral genes modulate the physiological machinery of infected cells that lead to cell transformation and development of cancer. One of the important adoptive responses by the cancer cells is their metabolic change to cope up with continuous requirement of cell survival and proliferation. In this review we will focus on how DNA viruses alter the glucose metabolism of transformed cells. Tumor DNA viruses enhance “aerobic” glycolysis upon virus-induced cell transformation, supporting rapid cell proliferation and showing the Warburg effect. Moreover, viral proteins enhance glucose uptake and controls tumor microenvironment, promoting metastasizing of the tumor cells.

## 1. Introduction

Development of cancer is a multistep process. Cancer cells differ from normal cells by genetic, metabolic, and histological features. Cancer cells have to fulfill their needs for continuous proliferation. Hence, they acquire various hallmarks during the process of tumor progression, such as self-sufficiency in growth signals, insensitivity to growth-inhibitory (antigrowth) signals, evasion of programmed cell death (apoptosis), limitless replicative potential, sustained angiogenesis, and tissue invasion and metastases [1]. 

Viruses play an important role in cancerogenesis. Globally, it is estimated that approximately 20% of all cancers are linked to infectious agents [2]. The viral genes transcribed or expressed in infected cells modulate the physiological machinery of cells that leads to cell transformation and development of tumor. One of the important adoptive responses by the cancer cells is their metabolic change to cope up with continuous requirement of cell survival and proliferation. In this review, we will focus on how DNA viruses alter the glucose metabolism of cancer cells during carcinogenesis.

## 2. DNA Tumor Viruses: An Overview

In 1960, Sweet and Hilleman discovered a new virus in cultures of kidney cells of rhesus monkeys, producing vaccines to poliovirus [3]. This virus was named Simian vacuolating virus (SV40). Two years later, the tumorigenic potential of this monkey virus was revealed [4]. At the same time, it was also shown that human adenoviruses could induce tumors in newborn hamsters [5]. For now, many DNA tumor viruses are known; they are grouped in four families, namely, SV40 and polyomavirus, papilloma viruses (HPV), adenoviruses, and herpesviruses. Because of their relatively small genomes and striking biological effects, it is generally assumed that DNA tumor viruses have evolved to target the minimal number of cellular nodes and pathways required for transformation. Studies of DNA viruses have led to the identification of viral genes responsible for cancer induction and paving the way to our current understanding of cancer at the molecular level [2]. In their life cycle, viruses replicate, inducing the cytopathic effect in the host cells and forming new viral particles. Herpesviruses are able to establish persistent infection transforming the host cells. HPV, adenoviruses, and polyomaviruses induce the host cell transformation while infecting nonpermissive cells and integrating into the host genome (see Table 1).

## 3. Glucose Metabolism in General

It is well known that tumor cells differ from normal cells by glucose metabolism. At the ordinary physiological conditions, one glucose molecule is converted into two pyruvate molecules. Pyruvate oxidation on mitochondria to CO_2_ and O_2_ results in synthesis of 38 ATP molecules per molecule of glucose [6]. When concentration of oxygen is diminished, no pyruvate oxidation is carried out. Pyruvate is converted to lactate; that is, anaerobic glycolysis is activated. This conversion produces NAD^+^, which is required for glycolysis. Glucose is also used by pentose phosphate pathway to produce nucleic acids and NADPH. NADPH is required for anabolic biosynthetic reactions as well as to neutralize ROS [7].

**Figure 1:**
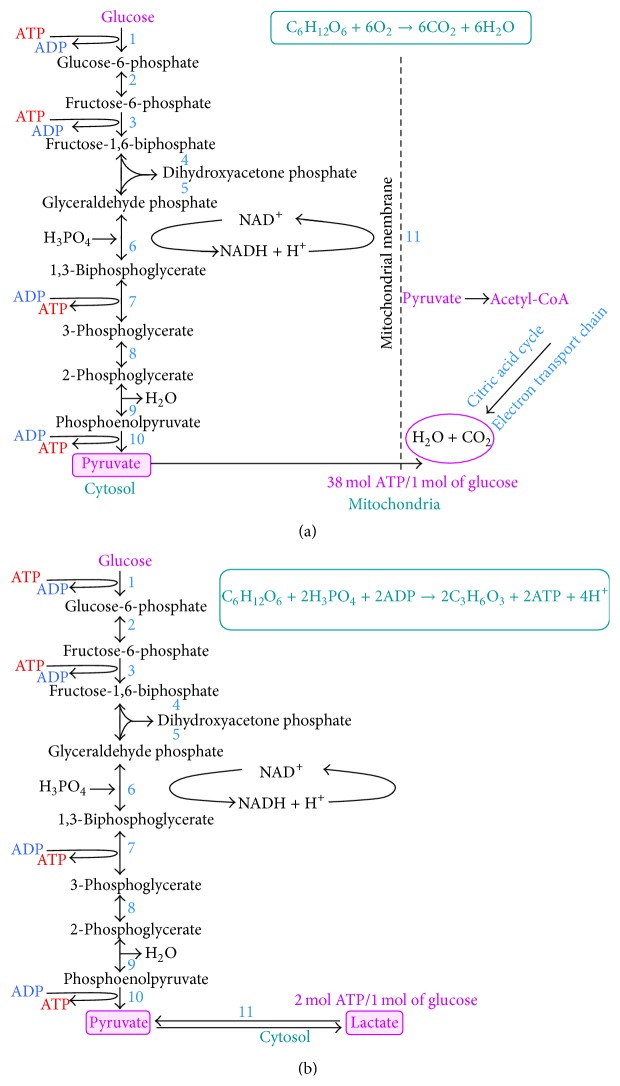
Glucose metabolism at the normal and hypoxic conditions. (a) Glucose is metabolized to pyruvate; the latter undergoes Crebb's cycle in mitochondria and catabolized to CO_2_ and oxygen, while 38 molecules of ATP are synthesized. (b) Anaerobic metabolism of glucose, resulting in lactate production and two molecules of ATP. No mitochondria are involved in this process. Cancerous cells use this way of glucose metabolism even at the normal conditions, that is, so-called “aerobic” glycolysis takes place.

Cells secrete lactate and produce only 2 ATP molecules during glycolysis as compared to pyruvate oxidation [8]. Noteworthy, cancerous cells under normal conditions (in the presence of abundant oxygen) still convert pyruvate to lactate, in parallel to pyruvate oxidation; that is, the Warburg effect is observed (Figures 1(a) and 1(b)). Despite the fact that only 2 molecules of ATP are produced as a result of so-called “aerobic” glycolysis, the rate of reaction is quite high, compared to ATP synthesis on mitochondria (at least nine reactions should be carried out).

Excess lactate production increases the acidity of tumor cell microenvironment and this favors the tumor cell invasion and metastasis [9]. Anaerobic glycolysis is used by tumor cells at hypoxic conditions, which is generally found in solid tumors due to deregulated vasculature. “Aerobic” glycolysis also provides the biosynthetic advantage for tumor cells. Glycolytic intermediates are utilized by proliferating cells to produce fatty acids and nonessential amino acids [10].

In addition to glycolysis, cancer cells exhibit increased gluconeogenesis, glutaminolytic activity, glycerol turnover, pentose phosphate pathway activity,* de novo* fatty acid synthesis, reduced fatty acid oxidation, and modified amino acid metabolism [11]. We have to emphasize that mitochondrial respiration is not hampered in cancer cells [12] but operates at low capacity [13].

Enhanced glucose uptake has also been exploited in FDG-PET technology used clinically for the tumor detection. There are a lot of studies devoted to target the metabolic pathways as anticancer therapy [14].

## 4. Regulation of Warburg Effect

Many oncoproteins and tumor suppressor proteins can affect the cancer cell metabolism [15]. Transcription factor HIF1A [16] and MYC oncoprotein [17] are involved in upregulation of glucose transporters and many enzymes involved in glycolysis. MYC can also promote the expression of PKM2, resulting in faster proliferation. Tumor suppressor p53 (TP53) can inhibit glycolysis by inducing TIGAR, a regulator of glycolysis and apoptosis [18]. This can support oxidative phosphorylation by inducing SCO2, which is necessary for the formation of electron transport chain [19]. Downstream signaling molecule of PI3 Kinase, AKT1, can enhance glycolysis by various ways. AKT1 promotes glycolysis by increasing expression and membrane translocation of glucose transporters. It also phosphorylates glycolytic enzymes, such as hexokinase and phosphofructokinase 2. AKT1 stimulates mTOR kinase, which activates transcription factor HIF1A even at the normoxic conditions [15]. Loss of AMPK signaling, which is inhibitor of mTOR, also stimulates glycolysis.

## 5. Virus-Encoded Proteins Play an Important Role in Regulation of “Aerobic” Glycolysis

### 5.1. Herpesviruses

EBV (HHV4) and KSHV (HHV8) belong to the Herpesviridae family. EBV is associated with BL, a highly aggressive malignancy that is developing from germinal center B-cells [20]. Characteristic of all BL subtypes is enhanced expression of MYC oncoprotein, due to chromosomal rearrangements [21, 22]. Recently we reported that in BL cell lines MYC is the main regulator of “aerobic” glycolysis, while in LCL, with the low expression levels of MYC, HIF1A controls the Warburg effect [23, 24]. HIF1 is a heterodimer consisting of oxygen dependent transcriptional factors HIF1A and ARNT, or HIF1B. The stability of HIF1A is regulated by oxygen level, while HIF1B is constitutively expressed. At the normoxic condition HIF1A is ubiquitinated by VHL (E3 ubiquitin ligase) at the specific proline residues (402 and 564) [25] that are hydroxylated by prolyl hydroxylases (PHDs) [26, 27]. The ubiquitinated HIF1A undergoes intensive proteasomal degradation. PHD enzymes require iron (Fe^2+^) and ascorbate as cofactors to perform hydroxylation [28]. At hypoxic condition oxygen is not available for hydroxylation of prolines that results in stabilization of HIF1A [29]. The stabilized HIF1A translocates to nucleus where it forms the HIF1A-ARNT heterodimer and activates transcription of a set of genes and also those involved in regulation of the Warburg effect.

We have reported earlier that even under normal oxygen level EBNA3 binds to PHD2 and EBNA5 to PHD1, thus, inactivating both enzymes. As a consequence, ubiquitination of HIF1A is inhibited, and the Warburg effect is activated [24].

Another herpes virus, KSHV, also promotes the Warburg effect. KSHV induces the expression of miRNA clusters that inhibit the expression of gene, encoding the HIF1A prolyl hydroxylase PHD1 and also the mitochondrial heat shock protein HSPA9 [30]. As a result, the HIF1A is stabilized and transactivates responsive genes. It was shown that one-third of the 194 different biochemicals were altered upon infection of endothelial cells with KSHV, compared with the noninfected host cells, using a metabolomics approach. Noteworthy, the number of altered metabolic pathways was similar to that observed for cancer cells. Pathways include amino acid metabolism and many glycolytic intermediates, such as 3-phosphoglycerate and 2-phosphoglycerate and phosphoenolpyruvate. The pentose phosphate pathway intermediates, such as ribose 5-phosphate, ribulose 5-phosphate, and/or xylulose 5-phosphate, were elevated significantly in KSHV infected samples. Metabolites involved in* de novo* fatty acid synthesis were also increased in KSHV infected cells. Moreover, inhibition of fatty acid synthesis resulted in induction of apoptosis in infected cells [31].

### 5.2. Polyomaviruses

JCV, SV40, and virus, obtained from a specimen of a renal transplant patient with initials B.K. (BKV), are common polyomaviruses in human populations.

The SV40 transformed rabbit chondrocytes showed alterations in the activities of mitochondria and metabolism. Increases in “aerobic” glycolysis and in activity of glycolytic enzymes were observed in SV40-transformed cells, probably due to chromosomal rearrangements induced by virus [32]. It was also shown, using transfections of primary human fibroblasts with large and small T antigens of SV40 in different combinations along with hTERT and HRAS, that the large T antigen expression leads to decreased dependency of transformed cells on mitochondrial energy production [33]. Noteworthy, the small T antigen of SV40 expression resulted in activation of the AKT signaling, enhancing “aerobic” glycolysis [34, 35]. Interestingly, medulloblastoma cells, expressing the large T antigen of JCV, showed significantly lower mitochondrial respiration and glycolysis. Upon glucose deprivation, T-antigen expression was suppressed due to activation of AMPK, an important sensor of the AMP/ATP ratio in cells. Therefore, the consumption of glutamine increased threefold in cells that expressed the large T of JCV [36]. As was mentioned above, TP53 can inhibit the Warburg effect [37]. It is well known that the large T antigen of polyomaviruses SV40 [38], JCV [39], and BKV [40] binds to TP53 and abolishes functional activity of the latter as transcription factor. Hence, functional inactivation of TP53 not only promotes cell transformation but also induces the metabolic switch.

### 5.3. Papillomavirus

It was shown that HPV encoded E2 protein is localized predominantly in the nucleus of infected cells. However, in the case of oncogenic (high-risk) strains 18 and 16 the E2 protein can shuttle between cytoplasm and nucleus. It was shown, using mass spectrometry of interactome, that cytoplasmic E2 is associated with the components of respiratory chain in the inner mitochondrial membrane. Electron microscopy showed that E2 alters morphology of cristae and enhances the production of mitochondrial reactive oxygen species (ROS). Such ROS release was found concurrent with stability of HIF1A and increased rate of glycolysis [41]. Another HPV-encoded oncoprotein, E6, also can promote the Warburg effect through inhibiting the binding between HIF1A and VHL. This abolishes VHL-mediated HIF1A ubiquitination, thus stabilizing the latter [42].

### 5.4. Adenoviruses

The ability of adenoviruses to perform the metabolic shift was demonstrated by infection of primary rat embryonic fibroblasts (REFs) with the oncogenic adenovirus type 12, in comparison with nononcogenic types 3 and 6. REFs, infected with type 12 virus, intensively used glucose at the ordinary conditions; both, “aerobic” glycolysis and pyruvate oxidation, took place. Similar metabolic switch was observed in the hamster sarcoma cells infected with type 12 adenovirus [43].

Recently it was shown that the adenovirus encoded oncogene E4ORF1 can induce* MYC* that plays an important role in glycolysis. Transcriptional activity of the MYC protein is enhanced by E4ORF1. Moreover, the expression levels of enzymes involved in “aerobic” glycolysis, such as hexokinase 2 (HK2), phosphofructokinase 1 (PFKM), GAPDH, and LDHA, are increased [44]. As was discussed earlier, these enzymes are encoded by the MYC-dependent genes.

## 6. Involvement of DNA Tumor Viruses in Glucose Transport

As was discussed above, glucose is the preferential source of energy for cancer cells; therefore, they need massive supply of glucose compared to normal cells [45]. By hijacking glucose transport system, DNA tumor viruses are able to deliver huge amounts of glucose for proliferating cells, enhancing their tumorigenic capacity [2]. Several viral proteins can facilitate the glucose transport in cancer cells.

Notably, the rise in glucose transport in cancer cells is not due to* de novo* synthesis of a delivery system but by alteration of already existing glucose transport system of cells [46]. Different hypotheses have been proposed to explain this phenomenon, including not sufficient glucose dephosphorylation dependent on glucose-6-phosphatase, increase of HK expression, and/or the overexpression of glucose transporter (GLUT) proteins [47].

GLUTs are a group of membrane proteins that facilitate the transport of glucose across the plasma membrane. Human genome encodes 14 isoforms of GLUT protein, and GLUT-1,-3,-4 and -12 are involved in cancerogenesis [48]. Expression of GLUT is under the control of activated HIF1A [49]. Infection with DNA tumor viruses leads to elevated expression of GLUT proteins, increasing the glucose uptake. We have shown earlier that expression of* GLUT-1* at mRNA level was induced in EBV positive LCLs and BL cell lines, compared with EBV negative cells [23, 24]. Upon latent infection of human monocytic cell lines with KSHV, GLUT1, and HK expression are increased at the protein level [50].

Not only are the levels of glucose transporter molecules elevated, the trafficking mechanism is also altered to ensure ample supply of glucose. Virus encoded proteins enhance the translocation of GLUT molecules to a cell surface; hence, there is another strategy to increase the glucose uptake. For example, AKT hyperphosphorylation upon KSHV infection correlates with plasma membrane exposure of GLUT1 [51]. Similarly, EBV also induces the translocation of GLUT1 via protein kinase IKKB-AKT pathway [52].

The HPV18-encoded protein E6 participates in stimulation of the SGLT1 activity. By this way, E6 accomplish cellular glucose uptake through Na^+^-coupled glucose transport mediated by SGLT1 [53]. It is noteworthy that in SV40 transformed mouse 3T3 cells hexose transporters are relocated from microsomal membranes to plasma membrane, suggesting that oncogenic DNA viruses utilize not only transcriptional regulation of glucose transport but also alterations in transporter trafficking during transformation [54].

## 7. Effect of DNA Oncoviruses on Secretion of Aerobic Glycolytic Waste

Lactate secreted into an extracellular matrix plays an important role in tumor metastasizing. This process is promoted by lactate-induced secretion of the hyaluronic acid by cancer-associated fibroblasts, thus generating an environment favorable for migration of tumor cells [55]. Moreover, lactate produced by tumor cells helps them to evade immune system by modulating dendritic cell activation and antigen expression that mediate the T cell responses [56, 57].

Activated T cells themselves use glycolysis as a main source of energy [58–60]. Importantly, the immune cells are struggling to get rid of lactate produced by themselves: cellular lactate transport depends on the ratio between the intra- and extracellular concentrations of lactate. Ultimately, leukocytes may be asphyxiated by lactate [61]. Cancerous cells of solid tumors ensure sufficient supply of nutrient and oxygen for rapid proliferation via lactate mediated upregulation of VEGF, thus inducing the angiogenesis [62]. Lactate stimulates the angiogenesis also via PI3K/AKT pathway [63].

The major transporter molecules of lactate in cells are monocarboxylate transporters (MCTs). MCT family consists of 14 members that are encoded by* SLC16A* gene family. The four MCTs (MCT1, MCT2, MCT3, and MCT4) are responsible for proton-linked transport of metabolically important monocarboxylates such as lactate, pyruvate, and ketone bodies [64–67]. MCTs carry 12 transmembrane domains with intracellular N- and C-termini and a large intracellular loop between transmembrane domains 6 and 7. MCT1 and MCT4 require a monotopic ancillary protein, CD147, for plasma membrane expression and function [68]. CD147 is a multifunctional glycoprotein expressed at higher levels by cancer cells and stromal cells in the tumor microenvironment [69]. KSHV-encoded latency associated nuclear antigen LANA either induces CD147 directly, binding to gene promoter, or transactivates* CD147* upon interactions with specificity protein 1 or early growth response protein 2 [70, 71]. Upregulation of MCT4 and CD147 has been also reported in HPV-induced squamous cell carcinoma of the uterine cervix [72]. Importantly, in BL cells MCT4 was also greatly upregulated [23].

**Figure 2:**
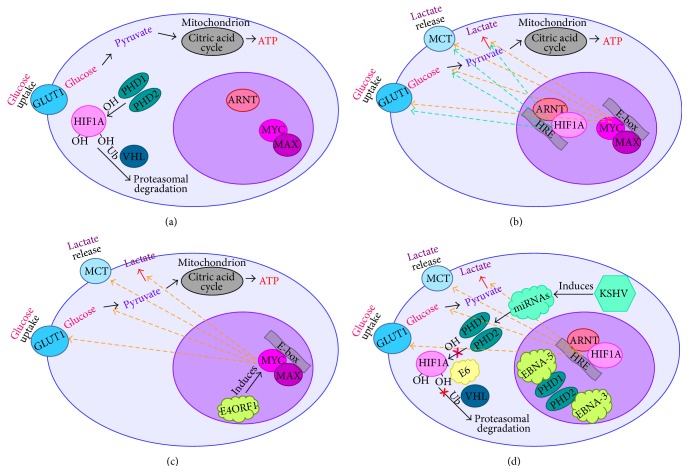
Tumor DNA viruses modify metabolism of the transformed cells. (a) Glucose is metabolized to pyruvate; the latter undergoes Crebb's cycle in mitochondria. (b) “Aerobic” glycolysis takes place, resulting in lactate production. HIF1A-ARNT and MYC-MAX heterodimers induce expression of a set of genes that are involved in glycolysis. (c) E4ORF1 encoded by adenoviruses induces MYC that lead to activation of glycolysis upon infection. (d) HPV-encoded E6 prevents ubiquitination of HIF1A by VHL protein; EBV-encoded EBNA-3 and EBNA-5 bind to PHD2 and PHD2, correspondingly, leading to inhibition of HIF1A hydroxylation; upon infection with KSHV a cluster of miRNAs is activated, resulting in inactivation of PHDs. This leads to stabilization of the HIF1A protein and, hence, to activation of “aerobic” glycolysis.

In conclusion, tumor DNA viruses modify metabolism of the transformed cells, supporting their rapid proliferation and showing the Warburg effect (summarized on Figure 2). Moreover, viral proteins enhance glucose uptake and controls tumor microenvironment, promoting metastasizing of the tumor cells.

## Figures and Tables

**Table 1:** Human tumor DNA viruses.

Family	Virus	Virus-cell interaction	Associated disease	Level of association %	Size of genome kb
Herpesviruses	Epstein-Barr virus, EBV	Episomal, rarely integrated in transformed cells	Endemic Burkitt's lymphoma (BL) AIDS-asscoiated lymphoma Nasopharengyal carcinoma (NPC)	98 100 100	172
	Kaposi sarcoma herpes virus, KSHV	Episomal, rarely integrated in transformed cells	Kaposi's sarcoma	97	165

Polyoma viruses	John Cunningham Virus, JCV	Episomal, rarely integrated in transformed nonpermissive cells	Progressive multifocal leukoencephalopathy	50–80	5.2
	Virus of B.K. patient, BKV	Episomal, rarely integrated in transformed nonpermissive cells	Nephropathy Nephritis Hemorrhagic cystitis	10–20	5.2
	SV40	Episomal, rarely integrated in transformed nonpermissive cells	Mesothelioma	10–20 cofactor	5.2

Papilloma viruses	HPV	Episomal, integrated in transformed cells	Cervical cancer	71–88 (types 16 and 18)	8

Adenoviruses		Integrated in transformed nonpermissive cells	Small cell lung cancer Childhood ALL	No data	35 kb (type 11)

## Abbreviations

AKT1: RAC-alpha serine/threonine-protein kinase 1
AMPK: Adenosine monophosphate kinase
ARNT: Aryl hydrocarbon receptor nuclear translocator
ATP: Adenosine triphosphate
BKV: BK virus
BL: Burkitt lymphoma
CD147: Extracellular matrix metalloproteinase inducer
E4ORF1: Early Region 4 Open Reading Frame 1
EBNA: EBV-encoded nuclear antigen
EBV: Epstein-Barr virus
FDG-PET: Fluorodeoxyglucose (^18^F) positron emission tomography
GAPDH: Glyceraldehyde-3-phosphate dehydrogenase
GLUT: Glucose transporter
HIF1A: Hypoxia inducible factor 1A
HK: Hexokinase
HPV: Human papilloma virus
HRAS: H-rat sarcoma
HSPA9: Heat shock protein-A9
hTERT: Human telomerase reverse transcriptase
IKKB: Inhibitor of nuclear factor kappa-B kinase subunit beta
JCV: John Cunningham virus
KSHV: Kaposi sarcoma herpes virus
LANA: Latency-associated nuclear antigen
LDHA: Lactate dehydrogenase A
MCT: Monocarboxylate transporter
mTOR: Mechanistic target of rapamycin
NAD: Nicotinamide adenine dinucleotide
NADPH: Nicotinamide adenine dinucleotide phosphate
PFKM: Phosphofructokinase 1
PHD: Prolyl hydroxylase domain-containing protein
PI3K: Phosphatidylinositol-4,5-bisphosphate 3-kinase
PKM2: Pyruvate kinase M2
REF: Rat embryonic fibroblast
ROS: Reactive oxygen specie
SCO2: Cytochrome c oxidase 2
SGLT1: Sodium-glucose transport protein 1
SLC16A: Family of proton coupled MCTs
SV40: Simian vacuolating virus
TIGAR: TP53-inducible glycolysis and apoptosis regulator
TP53 or p53: Tumor suppressor p53
VEGF: Vascular endothelial growth factor
VHL: Von Hippel–Lindau.
